# Action potential initiation in damaged axon initial segment

**DOI:** 10.1186/1471-2202-15-S1-P135

**Published:** 2014-07-21

**Authors:** Louis Jacques, Catherine E Morris, André Longtin, Béla Joos

**Affiliations:** 1 Department of Physics, University of Ottawa, Ottawa, Ontario, Canada, K1N 6N5; 2 Neurosciences, Ottawa Hospital Research Institute, Ottawa, Ontario, Canada, K1H 8M5

## 

The effects of physical trauma to nerve cells are of great interest in neuroscience and neuromedicine. The ion channels kinetics are affected by the trauma-induced damage to neural membrane structure. The density of affected channels is at its highest in the nodes and the axon initial segment (AIS). In this study we focus on the consequences of modifying sodium channels kinetics in the AIS to model the effect of damage. It is inspired by a similar study on damaged nodes of Ranvier [1] and by experiments done *in vivo* on AIS [2,3]. The AIS and the nodes of Ranvier have similar mixtures of channels, but differ in their shapes. Whereas the nodal channels are clustered at high density near a large volume of channel free low capacitance membrane the AIS is long with varying density profiles for each ion channel types. The AIS is the location for action potential (AP) initiation.

A neuron was simulated as three sections, each containing multiple compartments: the soma, the initial segment and the myelinated axon. Each compartment was modeled by the classical Hodgkin-Huxley equations using appropriate densities for each type of ion channel. The types of channels and their distributions were based on a previous study looking specifically at cortical pyramidal neurons having Nav1.2 and Nav1.6 at the AIS [4]. The kinetics were modified according to the coupled left-shift (CLS) model [1,5]. This model is based on observations showing that in damaged membranes Na channel kinetic midpoints shift leftward to more hyperpolarized values, effectively rendering the channels hypersensitive. In this study we explore the impact of CLS on the generation of APs in the AIS. The simulation includes NaK-ATPase pumps and inner and outer ion concentrations are monitored. With increased damage, the greater ionic conductance at rest potential (Na-leak conductance) puts greater strain on the pumps. In the case of a single node, hyperexcitability, tonic firing, burst firing, or inexcitability is observed for increasing values of CLS.

We explored the effect of damage on the initiation and propagation of APs during repeated stimuli which deplete concentration gradients. Our model allows the investigation of the effect of CLS on the location and duration of the initiation and the threshold duration of Ap-initiatingcurrents. We find that the current threshold for action potential initiation decreases with increased damage or decreased length of the AIS.
